# PREDICTORS OF DIGITAL COMPETENCE AMONG OLDER ADULTS INTERESTED IN AN INTERGENERATIONAL TECHNOLOGY PROGRAM

**DOI:** 10.1093/geroni/igad104.1717

**Published:** 2023-12-21

**Authors:** Skye Leedahl, Cindy Tsotsoros, Alexandria Capolino, Josie Santilli

**Affiliations:** University of Rhode Island, Kingston, Rhode Island, United States; University of Rhode Island, Kingston, Rhode Island, United States; University of Rhode Island, Kingston, Rhode Island, United States; University of Rhode Island, Kingston, Rhode Island, United States

## Abstract

The Rhode Island Office of Healthy Aging initiated a digiAGE collaborative. As a major part of this initiative, the University of Rhode Island launched the Engaging Generations: Cyber-Seniors digiAGE iPad Program and research project. We analyzed participants who took part in the study in 2021 and 2022 (N=324; age range=50-92; 77.2% female, 22.5% male; 65.1% White, 16.4% Hispanic, 11.4% Black, 4.9% Native American or Alaska Native, 1.2% Asian, 1.9% Multiple races; 83.6% English, 15.4% Spanish as primary language). In this study, we examined demographic characteristics (e.g., income, marital status, employment status, primary language, living alone status, education, gender, race/ethnicity, and Wifi access), and social well-being characteristics (e.g., depression, social isolation, loneliness, quality of life) as predictors of digital competence using structural equation modeling. Overall, this model showed excellent fit (𝜒2(53, N=324)=104.29, p<.05, with CFI = .96, TLI = .92, RMSEA = .06,CMIN/df = 1.97). The model indicated several direct and indirect effects. For indirect effects, income status predicted depressive symptoms, depressive symptoms predicted quality of life, and quality of life predicted digital competence. For direct effects, having access to a wireless internet connection within the home and education level predicted digital competence (Adj. R Square = 0.112). As technology becomes increasingly integrated into our daily lives, examining predictors of digital competence among older adults is important to identify whom to target for digital inclusion initiatives and how to provide support appropriately.

